# Raising a child bereaved by domestic homicide: caregivers’ experiences

**DOI:** 10.1080/20008066.2025.2463277

**Published:** 2025-02-20

**Authors:** Eva Alisic, Arend Groot, Hanneke Snetselaar, Tielke Stroeken

**Affiliations:** a Melbourne School of Population and Global Health, University of Melbourne, Melbourne, Australia; b Psychotrauma Centre Wilhelmina Children’s Hospital, University Medical Centre Utrecht, Utrecht, The Netherlands

**Keywords:** Bereavement, co-victims, domestic homicide, femicide, foster care, grandfamilies, grandparents, intimate partner violence, kinship care, traumatic grief, Duelo, co-víctimas, homicidio doméstico, femicidio, acogida temporal, familias de abuelos, abuelos, violencia de pareja, cuidado de parentesco, duelo traumático

## Abstract

**Background:** Optimising support for children and families affected by fatal family violence requires understanding all aspects of their experience. So far, little is known regarding the views of those who provide a home to children bereaved due to parental intimate partner homicide.

**Objective:** The aim of the current study was to provide an in-depth exploration of the experiences of caregivers raising children after the loss of a parent due to intimate partner homicide.

**Method:** Within the context of a mixed-methods study among 22 caregivers (16 female, 6 male, aged 33 to 71 years old) related to 35 children and young people (19 female, 16 male), bereaved due to parental intimate partner homicide in the Netherlands, we conducted a reflexive thematic analysis of the qualitative caregiver interviews.

**Results:** Based on caregivers’ accounts, we conceptualised four interrelated and ongoing challenges: (1) bringing the children into the family fold; (2) dealing with the perpetrator and relatives; (3) managing underprepared services; and (4) enduring it, mentally and physically. Sticking with their commitment to the children despite these challenges, caregivers also pointed to the potential for positive outcomes or turns of events, and recounted experiences of finding or making meaning.

**Conclusions:** The complexity of the challenges the caregivers in our study faced and their remarkable commitment and perseverance underscore the importance of concerted, continuing efforts to understand and respond to families’ needs in the aftermath of parental intimate partner homicide. We discuss practical implications regarding caregivers’ assessment of children’s needs, mental health care, information provision and agency, mediation of family conflict, provision of respite care, addressing financial and practical needs, and long-term and equitable access to support. We also propose a research agenda involving evaluation of current protocols, in-depth qualitative research, quantitative analyses (where possible based on pooled data), and intervention studies.

## HIGHLIGHTS

- Placement and caregiving decisions in the aftermath of a parental intimate partner homicide reverberate in both children’s and caregivers’ lives throughout the years.
- Caregivers were faced with complex and ongoing challenges related to home life, contact with the offender and relatives, and assistance by underprepared services.
- Caregivers expressed a strong commitment, ‘sticking with’ difficult family or service relationships for the sake of the children.

## Abstract (es)

**Antecedentes:** Para optimizar el apoyo a los niños y las familias afectadas por violencia familiar fatal es necesario comprender todos los aspectos de su experiencia. Hasta el momento, se conoce poco sobre las opiniones de quienes brindan un hogar a los niños que han sufrido un duelo por homicidio de uno de los padres por violencia de pareja.

**Objetivo:** El objetivo del presente estudio fue proporcionar una exploración en profundidad de las experiencias de los cuidadores que crían niños después de la pérdida de uno de sus padres debido a homicidio por violencia de pareja.

**Método:** En el contexto de un estudio de métodos mixtos entre 22 cuidadores (16 mujeres, 6 varones, de 33 a 71 años de edad) relacionados con 35 niños y jóvenes (19 mujeres, 16 varones), en duelo debido al homicidio parental por violencia de pareja en los Países Bajos, realizamos un análisis temático reflexivo de las entrevistas cualitativas a los cuidadores.

**Resultados:** Basados en el relato de los cuidadores, conceptualizamos cuatro desafíos interrelacionados y actuales: (1) incorporar a los niños al seno familiar; (2) lidiar con el agresor y los familiares; (3) gestionar servicios mal preparados; y (4) soportarlo mental y físicamente. Los cuidadores que cumplieron con sus compromisos con los niños a pesar de estos desafíos, también señalaron el potencial de resultados positivos o giros de los acontecimientos, y relataron experiencias de búsqueda o construcción de significado.

**Conclusiones:** La complejidad de los desafíos que enfrentaron los cuidadores en nuestro estudio y su notable compromiso y perseverancia subrayan la importancia de los esfuerzos continuos y concertados para comprender y responder a las necesidades de las familias después del homicidio de uno de los padres por violencia de pareja. Discutimos las implicancias prácticas en relación con la evaluación de las necesidades de los niños por parte de los cuidadores, la atención de la salud mental, la provisión de información y la capacidad de acción, la mediación de los conflictos familiares, la provisión de atención de relevo, el manejo de las necesidades prácticas y financieras, y el acceso a apoyo equitativo y a largo plazo. También proponemos una agenda de investigación que involucre la evaluación de los protocolos actuales, investigación cualitativa en profundidad, análisis cuantitativo (cuando sea posible basados en datos agrupados), y estudios de intervención.

## 1. Introduction

It is difficult, if not impossible, to overstate the impact of fatal family violence on those who are left behind. Our research team has been concerned with the impact on the children involved and the people who care for them. This paper focuses on the experiences of caregivers of children who lost a parent due to intimate partner homicide.

Globally, there has been an increase in policy attention to the number of intimate partner homicides, in particular intimate partner femicides. In 2022, the UN registered approximately 48,800 women and girls who had been killed by their intimate partners or other family members worldwide (United Nations Office on Drugs and Crime & UN Women, 2023). Considering the limited availability of data and the sometimes narrow definitions of homicide, these figures are likely underestimates (see e.g. Cripps, 2023). Generally, the data recorded on intimate partner homicides do not include how many children were bereaved or otherwise affected, but their numbers are likely significant (cf. an estimate of at least 55,000 children per year globally; Alisic, Krishna, et al., 2015).

There is some information available on what bereaved children are confronted with *before and during the homicide*. In the 80s and 90s, Dora Black and colleagues conducted seminal clinical work and research with children in the UK (Black et al., 1992; Black & Kaplan, 1988). Up to publishing the second edition of their book, they had seen 425 children, of whom 90% lost their mother at the hands of their father, and specifically analysed the data of 95 children referred to their clinic before 1993 (Harris-Hendriks et al., 2000). In two-thirds of the families, there had been long-standing conflict between the parents, with many children witnessing violence. At least a third of the children saw or heard the homicide. This aligns with more recent findings by Stanley et al. (2019) who scrutinised domestic homicide reviews (DHRs) completed in England and Wales for information about the children involved, as well as findings from a US-based study among 146 children bereaved by femicide, which reported an even higher rate of exposure to the homicide or the victim’s body (72%; Lewandowski et al., 2004).

Our team conducted a population-wide study of children bereaved by intimate partner homicide in the Netherlands between 2003 and 2012 (Alisic et al., 2017). A total of 256 children had lost a biological parent in this period on a population of approximately 16 million inhabitants, due to 137 cases of intimate partner homicide (88% femicides). We found that for at least 75% of the children, the homicide happened in their home, that over half of the children were at the location of the homicide when it happened (with varying degrees of certainty to the extent of exposure), and that the weapon used was most often a cutting weapon, followed by firearms and strangulation, suggesting frequent graphic aftermaths. For 83% of the children, there had been a suspected or confirmed prior history of violence or neglect in the home. Two-thirds of the children were younger than 10 years old at the time of the homicide, which is relevant in terms of their capacity to understand death and verbalise emotions, and the likelihood of challenges for adults around them to inform and involve them in subsequent decisions that shape their lives (cf. Callaghan et al., 2017; Convention on the Rights of the Child; United Nations, 1989).

The *impact* of parental intimate partner homicide on children involves all aspects of their lives, with key elements being having to deal with loss, grief and trauma (see Alisic, Krishna et al., 2015 for an overview); guilt and stigma (Eastwood et al., 2024; Marinkovic Chavez et al., 2024); and changed dynamics at home, in relation to relatives (including the perpetrator in many cases), at school and in the broader community (e.g. Alisic et al., 2018; Pitcho-Prelorentzos et al., 2023; Steeves & Parker, 2007). With regard to mental health impact specifically, out of the 95 children referred to the UK. Traumatic Stress Clinic mentioned above, almost 60% experienced behavioural problems, 25% had mild or isolated symptoms of posttraumatic stress, 25% had moderate to severe posttraumatic stress disorder (PTSD), and 40% showed symptoms of emotional disorders (Harris-Hendriks et al., 2000). Only 15% of the children had maintained their pre-homicide level of school performance. Lysell et al. (2016) conducted a rare study in which they matched 494 intimate partner femicide-bereaved children with children in the general population in Sweden and followed them up longitudinally for up to 37 years. The bereaved children had elevated risks of severe mental health difficulties, substance use disorders, violent crime offences and self-harm. While these studies show that various risks are elevated, they also show strong diversity in children’s outcomes.

As Stanley et al describe, ‘the most immediate impact for most of the children … was that their living arrangements changed following their mother's death’ (p. 65). In the US study by Lewandowski et al. (2004), 87% of the 146 children moved from their home after the homicide to live mostly with their mother’s relatives, followed by the perpetrator’s relatives, family members from both sides (when siblings were split up), and other caregivers, such as foster parents. Similarly, Harris-Hendriks et al. (2000) reported on their sample of 95 children that more than half went to live with relatives straight after the homicide: 35% with the mother’s family, 17% with the father’s family, usually the grandparents. About a third of the children went to foster-parents, ten children from three families went into residential care, and a few children went to a friend of the family or neighbour. A subsequent survey among referrers of the children, providing data on 61 children, showed that the children experienced many changes of home and carer, which is also a concern in the broader literature on children in out-of-home care (e.g. Liming et al., 2021; Vanderwill et al., 2021). Of those living with relatives after the homicide, the fewest changes of carer occurred when they were initially placed with relatives of the victim, rather than relatives of the perpetrator, where they were more likely to return to their surviving parent (Harris-Hendriks et al., 2000).

While the field of fatal family violence is gradually building an understanding of the circumstances and outcomes of bereaved children, little is known of their *caregivers’ own experiences*. Given that the stable presence of an adult can positively influence the long-term adjustment of children, understanding and responding to the needs of caregivers is essential for supporting children after intimate partner homicide (cf. Asif, Breen & Wells, 2024; Hardesty et al., 2008). Hardesty and colleagues are among the few exceptions who have looked into this. They conducted telephone interviews with 10 adults in the East and Southwest regions of the US, who were primary caregivers or otherwise actively involved in the children’s care following a femicide. In all but one of the families, children moved to new homes and communities after the homicide. The interviews, conducted between 5 weeks and 5 years post-homicide, conveyed ‘a unique pileup of stressors and hardships that taxed their ability to adjust’ (p. 122) including other deaths and illnesses in the family and among caregivers themselves, a diversity of trauma-related needs among the children, financial strains and family conflict.

Findings such as these are important for policy and practice interventions to better assist families. To contribute to this area, the aim of the current study was to conduct an in-depth exploration of the experiences of caregivers who look after children bereaved by parental intimate partner homicide.

## 2. Methods

This qualitative study was conducted in the context of a larger mixed-methods project on the characteristics, circumstances, and wellbeing of children who had been bereaved by parental intimate partner homicide in the Netherlands (see Alisic et al., 2014; Alisic, Groot, et al., 2015; Alisic et al., 2017; Alisic et al., 2018).

The Netherlands is a high-income country (World Bank, 2024) and often scores in the top 20 countries on child rights and youth development (e.g. Kids Rights Index, Youth Progress Index; Kids Rights, 2024 and Harmacek et al., 2023). It has structures in place for child and adult community services as well as specialist mental health care, although access is not homogeneous (see e.g. Pannebakker et al., 2024, p. 388). The study was approved by the University Medical Center Utrecht Ethics Committee (13/609).

The interviews were conducted in 2014. Broad stroke findings regarding caregiver views on children’s outcomes were reported in a government-commissioned Dutch report (Alisic et al., 2014), whereas we provide a much more in-depth analysis here, focusing on caregivers’ own experiences. This analysis took time, due to the nature of the material, but also because of multiple job changes, serious illnesses among research team members and their loved ones, a pandemic, and academic and clinical ‘life’ happening. Our ongoing research and new interviews in Australia and the UK (Alisic et al., 2023; Kurdi et al., 2024) suggest that the findings of this paper remain just as relevant and important for practice however, and considering the burden for people with lived/caregiving experience, it appears important to maximise learning from existing material, which is why we – finally – share these results from our Dutch interviews here.

### 2.1. Participants

Eligible for this study were caregivers of children and young people (up to 25 years of age) who had lost a biological parent due to parental intimate partner homicide while they were a minor. We recruited potential participants via multiple routes (see Alisic, Groot et al., 2015 for details, which also provides an indication regarding the services available at the time). One route was via the client database of the Psychotrauma Centre of the Wilhelmina Children’s Hospital, a nationwide tertiary mental health care provider with specialist expertise regarding domestic homicide, where our team was based. We also invited potential participants via our collaborating partners (e.g. the Child Care and Protection Board, youth services). Finally, we asked associations of professionals and victim support associations to distribute information about the study (e.g. via their newsletter), inviting people to contact us. After informing potential participants of the project and its procedures, we obtained written consent if they wanted to go ahead with the study. If they took care of children without being their legal guardian, we also obtained written consent from the guardian before conducting the interview. We gave participants a 10-euro voucher to thank them for their participation (this was not mentioned during the informed consent procedure).

In total, 22 caregivers (16 female, 6 male, aged 33 to 71 years old), related to 35 children and young people (19 female, 16 male), participated in the mixed-methods study. The caregivers were grandparents (9 participants), other family members (7 participants, including 1 offending parent who had been released from prison), friends of the parents (4 participants), or unrelated to the children (2 participants). The children were 7 to 24 years old at the time of the interviews and had been 4 months to 17 years old at the time of homicide. The homicide had happened 12 months to 18 years prior (*M* = 7.6 years, *SD* = 4.6 years). All children had been born in the Netherlands, although for 7 of them (20%), at least one parent had been born abroad. Most of the children (*n* = 32; 91%) had lost their mother. For 31 children, we had information on their whereabouts at the time of the homicide: 9 children witnessed the homicide or the crime scene, 14 were ‘on location’ but their exact exposure was unclear, and 8 were certainly not where the homicide happened.

Out of 19 participating caregivers who completed the Dutch RAND-36 Health Survey (Zee et al., 1996) regarding their quality of life, 11 (58%) experienced difficulties on at least one subscale (most often related to energy/vitality, followed by social functioning and mental health, while physical functioning was least affected). Of 18 participants who were related to, or friends with, the parents of the children before the homicide and completed the Dutch Inventory of Traumatic Grief (Boelen et al., 2003), 5 (28%) showed clinical levels of grief. On the Dutch Family Home Environment Scale (Van der Ploeg & Scholte, 2008), 14 of the 18 participants who completed the scale (78%), experienced mild or substantial difficulties (28% and 50% respectively). These difficulties had mostly to do with communication with the child and the relationship with the other caregiver, and least with responsivity of the caregiver to the child.

### 2.2. Interviews

Our interviewers were qualified mental health professionals (master’s degree level; HS, TS, AG). The interviews took place at participants’ homes and were semi-structured, guided by a topic list (see Boeije, 2010). The questions were based on a review of the literature (e.g. Hardesty et al., 2008; Harris-Hendriks et al., 2000; Steeves et al., 2011; Steeves & Parker, 2007; Van Nijnatten & Van Huizen, 2004). The items covered the children’s psychosocial development, placement, contact with the perpetrating parent, custody/guardianship, family circumstances and interactions, the role of professional organisations, helping factors, and contact with people who had also lost a family member due to homicide. All interviews took place in Dutch and were audiotaped, although the recorder crashed during one interview, which we included based on notes taken during and directly after the conversation. The length of the interviews ranged from 27 to 69 min (*M* = 41 min; *SD* = 12 min).

### 2.3. Analyses

We transcribed the interviews verbatim. Our analysis was predominantly thematic. We initially summarised each interview with regard to aspects centred on the young person: placement, contact with the perpetrating parent, guardianship, role of services, role of the family, helping factors, identity and future, and psychological wellbeing (AG, HS, TS, on Excel spreadsheets). We synthesised these summaries per topic, exploring similarities and differences across the individual summaries (EA, AG, HS, TS) and where necessary going back to the original interviews. After this, EA went back into the interviews and conducted line-by-line coding, synthesis, and looking at patterns and throughlines in line with reflexive thematic analysis (Braun & Clarke, 2006, 2019, 2023). We discussed the descriptions of the developed themes (EA, AG) and EA finetuned these accordingly. Relevant quotes were translated to English once the analysis had been finalised. We concentrated our analysis on elements that caregivers shared about children in relation to themselves or family life. For example, a note on a child having nightmares would not feature, but a note on a child’s nightmares waking up the family or having the caregiver worried would.

Regarding our positionality, we are a research team consisting of clinician-researchers whose main studies and disciplinary contexts involved psychology, social work and public health, with Dutch autochthonous and bi-cultural/migration roots, from middle-class, middle-to-highly educated, mostly privileged backgrounds. We care about equity even though we see the opposite of equity playing out in our clinical and/or research work on a regular basis. We have experience working with families in the aftermath of an intimate partner homicide, but we do not have our own lived or caregiving experience in the context of this type of traumatic loss. We have taken caregivers’ accounts mostly at face value, while acknowledging that we would not be able to capture ‘truth’, since both interviewees and researchers bring their own interpretations and lenses. In other words, we have taken a critical realist approach (Terry et al., 2017). We do occasionally – gently – question caregivers’ accounts in our results, specifically when we recognised that they all, irrespective of their role/relationship with the children, felt that their type of arrangement was best for any child bereaved by parental intimate partner homicide.

Overall, reflexivity was integral to our research from its design onwards. Key moments where we practiced reflexivity included regular team conversations about our positionality and emotional responses to the work (e.g. regarding collecting data, engaging with families, reading case files and listening to the accounts), EA writing memos throughout the analysis process, EA and AG holding peer supervision meetings regarding the evolving content of the analysis, and EA engaging with people with lived and caregiving experience in the context of a broader international team investigating the impact of intimate partner homicides on young people.

## 3. Results

Our analysis converged on four key, interrelated challenges: (1) bringing the children into the family fold, (2) dealing with the perpetrator and relatives, (3) managing underprepared services, and (4) enduring it, mentally and physically (see Box 1). Within the context of these challenges, caregivers also pointed to experiences of positive change and meaning. We start each challenge section with a brief overview paragraph. While these challenges came up across many caregivers’ narratives, there was also variety; we touch on exceptions where we can.

Box 1. **Caregivers’ key challenges.**

Challenge 1: Bringing the children into the family fold.

Challenge 2: Dealing with the perpetrator and relatives.

Challenge 3: Managing underprepared services.

Challenge 4: Enduring it, mentally and physically.

### 3.1. Challenge 1: bringing the children into the family fold

Caregivers were literally tasked with the challenge of integrating one or more children in their existing home life. This involved deciding to look after the children, making financial and practical sacrifices, adapting to each other’s characters and preferences, and responding to children’s emotional needs.

The first step was the decision process itself. In some cases, it was a natural progression, for example when caregivers had already offered respite care a few days per week or during the holidays. In other cases, caregivers had to plead with services to be allowed to take in the children, especially when there was conflict within the family about who should – or should not – look after them. When caregivers had children living at home, the decision process could involve considering whether they got along well with the bereaved children or had any concerns. For example, one child worried about their parents’ ability to cover their study costs if other children joined the family.

Caregivers generally expressed that their placement was the best option, or at least had advantages. For example, a grandmother (mother of the victim) felt that grandparents can really incorporate a child’s mother in the child’s life, which unrelated foster parents cannot do. Comparing families in a peer support group, she had the impression that children in foster families were taught more avoidance as a result. In contrast, a foster parent felt that their neutrality, not having a particular loyalty, helped children’s contact with family members from both sides. Moreover, an advantage of family not being involved was being able to raise the children as one sees fit, as one’s own children. Caregivers who were relatives disputed that however, and thought that a child is better off with family – literally providing familiarity as well as continuity – than with unknown foster parents. In turn, the perpetrator-caregiver asserted that his child had developed normally and did not have any mental health difficulties, implying that it was a successful living arrangement for the child. However, another family described a difficult situation where a placement with a perpetrator affected subsequent home dynamics:

When they returned, after the placement, they were completely upset, distraught. Father had really lectured them that we had done everything wrong, that they shouldn’t be kind to us. So it was very difficult, we just left the children be and thought it would sort itself naturally. Eventually, things did work out. But for the children it was very confusing. [INV10]

Taking in one or more children came with financial and practical sacrifice. Examples included caregivers who gave up or reduced their employment to be able to be with the children, renovated or extended a home to accommodate everyone, or relinquished plans such as extended travel or a quiet retirement (we return to this in challenge 4). Substantial sacrifice came also from the children’s side, for example when siblings had to be separated due to space limitations or tensions that were just too big to sustain (although there was also a situation where this meant more calm for one of the children), which affected caregivers as well. Specifically, children were also seen to be focused on making sure that caregivers who were family members were doing okay:

Yes it’s obviously quite difficult for them too, since you’re living with grandpa and grandma, you know they are grieving. It’s very mixed of course. [INV7]

Learning to live together and adapt to each other’s characters could be a demanding undertaking, sometimes done with the support of counselling or specific strategies to get to know each other. One such strategy was ‘children’s meetings’ to discuss what children missed from home, what home rituals they previously had (e.g. for birthdays) and what new rituals they would like. Several caregivers talked about ways in which they tried to help the children feel at home. This could include getting organic food for children from an eco-minded family, trying to find shampoo that smelled like what children originally had or making their room look similar to their old one. However, one caregiver explicitly said it was important not to try to replace the deceased mother but ‘rather be a good fakie’ and, similarly, not to compare the new child to their own children. She also suggested that some friction was unavoidable and healthy:

Then he also started to really feel at home and clash a bit more with his [new] brothers. That was all okay, we were only happy about that. [INV9]

When children were split, this could create some specific new dynamics. For example, a younger sibling suddenly became the oldest child in his new family. Some caregivers deliberately tried to work towards shared experiences despite siblings being separated:

We'd all go to the home where one of them had their birthday in our pyjamas in the morning. [INV12]

Occasionally, caregivers talked of children developing incorrect, romanticised views of their family life pre-homicide, or that, had they still been with (mostly) mom, there wouldn’t be the rules or limitations they experienced in their current family.

Living together brought the complexity of supporting a child who had been exposed to traumatic grief and, in several cases, to a dysfunctional household beforehand, or to attachment ruptures due to placement changes afterwards. Understanding and responding to children’s emotional needs was a prominent part of participants’ accounts. One couple explained that the young girl in their care had such severe anxiety that she had bitten all sides of her baby bed and couldn’t be left alone at all until they had gradually worked with her towards understanding that she wouldn’t need to leave, and that even if she was naughty, she would be allowed to stay with the family:

So we’ve tried to gradually build that up, to the girl she is now […] She knows what every day will bring, we talk that through. And that aspect of safety [helps], that we tell her again and again that she will continue to live with us, even if she’s a little naughty, that she won’t need to leave. And her cousins, her sisters in a way, they also confirm that for her, they also say ‘you’re my sister and you’ll continue to stay here, and we want to do fun activities with you as sisters.’ [INV6]

This explicit expression of commitment to the child, conveying that the placement was not a short-term solution, came up in several interviews, including one with a caregiver who had observed that the child was constantly trying to please family members out of fear of losing the placement.

One of the difficulties related to responding to children’s emotional needs was when and how to tell the children about the homicide. Caregivers’ assessment of children’s readiness for this naturally seemed to be influenced by their own norms, skills and emotional state. A few children had been informed of the death of their (mostly) mother early on, but not of what had happened until years later. Across families, the ‘homicide story’ appeared to have three to four elements, that were often shared separately over time: (a) that mother was dead, (b) that it was a homicide, (c) that (in most cases) father was the perpetrator, and (d) how the homicide had happened. Some caregivers indicated that they had told the story over time guided by the child’s questions and/or relevant external circumstances. For example:

Then came the moment that father was granted early release, and she was actually too young to know the truth. But, we had to tell her because father is very narcissistic person. So if he was to seek contact with her, then she should know the ins and outs and how do I need to respond […] So she knew that her father had killed her mother, but she kept searching on the computer. She had found photos on the computer, of the father, of the mother, and so also the piece detailing exactly what had happened that evening. And then I came to an agreement with her that she wasn’t allowed to read that because I felt she wasn’t ready for it yet, and we agreed with her that we would read and discuss it together during the Christmas holidays. She did wait patiently until the Christmas holidays. So now she actually knows in detail that it happened through knife stabs, that mother was taped down, you name it. [INV6]

### 3.2. Challenge 2: dealing with the perpetrator and relatives

Perpetrators took up a vast amount of emotional and practical energy of caregivers, either directly or indirectly. Our interviewees overwhelmingly described situations of perpetrators overstepping boundaries, frustrating children’s lives, posing risks for future harm or showing a painful disdain for the children or caregivers themselves. They dealt with these situations in a range of ways, from pushing back to tolerating what was legally required. In addition, caregivers had mixed experiences with other family members, often involving conflict, occasionally involving cooperation.

Multiple perpetrators did not adhere to contact rules, sending inappropriate cards, gifts and letters, and regularly trying to circumvent communication restrictions in general. For example, one sent gifts as if they came from the grandparents. Another called when he knew caregivers would not be at home and pressured the caregivers’ children to pass the phone on to his child. Yet another made use of a change in professional guardians to send a letter with her phone number on it, which was intercepted by an alert foster parent:

There was a guardian on maternity leave, and then we had a very experienced gentleman who had been doing it for years, and still, the mother … something slips through where the child is emotionally manipulated, or a letter arrives saying: you can always call me with a mobile number. Luckily, I had read it first before it reached [Child], where the parents are so cunning to bypass the rules as soon as there's a new guardian. Yes, then it's good that you're an experienced foster family, that you immediately call out: wait a minute. [INV22]

Beyond this, children’s, and therefore, caregivers’ lives were frustrated by perpetrators in various ways. For example, one father did not let children get access to important personal items. Another would frequently cry in front of the children during prison visits, suggesting that *he* was the victim and making the children feel concerned about his well-being, which angered the caregivers. A third had had phone contact ordered by youth services on Saturdays, which meant for the family that that day revolved around the perpetrator.

Several caregivers were concerned about ongoing risks of harm. This included fears of kidnapping by the perpetrator or ‘the other side’ of the family, fears of the perpetrator’s intent to kill the children, and fears of intimidation by the perpetrator upon weekend parole or eventual release, for example of the perpetrator suddenly showing up at one of the children’s activities. These risks put caregivers on continuous alert. Several sought to get information about possible prison release dates, which were hard to obtain. One installed cameras around the house, while another insisted on the children carrying a phone with them:

When I heard he was free, I was really looking at every car that came down the street, so to speak. And I didn't let them leave the house without a mobile phone anymore. [INV14]

Caregivers also indicated pushing back when boundaries were overstepped, sometimes with the help of services as a buffer, sometimes on their own, and sometimes against services’ suggestions. Other responses included maintaining a fairly neutral approach or limiting any concessions to the perpetrator to what was minimally required in a legal sense. One caregiver explained her stance as follows:

Honestly, I do a lot of things that aren't allowed. I'm a bit of a stubborn woman. I can't help it. We're the ones getting our hands dirty, you know. He's not going to tell me what to do, I really hate that. So they had to get used to us quite a bit. [INV4]

In another instance, caregivers described how they had kept the door open for the perpetrator to maintain contact with the children because they had been on good terms previously, but were confronted with how father ignored the children and deemed the caregivers not good enough. They described it as hurtful how a parent can be able to abandon their children:

The two eldest experienced all those legal proceedings, and I really, really wanted to spare them from that. […] they had to listen to all of that, and your father sitting there not even looking at you, and … well, I found that very tough. For them, not for me. It doesn't matter to me if he walks past me, but for them, I found that … it hurt me a lot. [… We were at the funeral of another family member] and we're sitting at a table, and he comes from over there and walks towards us. I think: oh, he's going to [Child]. He walks past her chair and goes to someone else. Well, then … then you get very sad and very angry. … when I see what wonderful children they are, and, and that you don't do anything with that, I find it a shame. But, I don't think he realises it, but I feel really sorry for the children. I really wanted to spare them that, only, yeah … sometimes, those things, you can't spare them, right? [INV8]

Only a small number of interviewees experienced perpetrator parents as showing some level of care: a neutral foster parent indicated that the perpetrator still had custody and cooperated as needed, a caregiver from the perpetrator side said that the perpetrator wanted the best for the child, and the caregiver-perpetrator himself suggested that nobody should end anyone’s life and that he was entirely in the wrong but also that one should see the action separate from the person. Other interviewees tended to disagree with the latter: several caregivers felt that a domestic homicide – when it was not out of self-defence – equalled giving up one’s rights to custody, and they conveyed strong emotions in cases where the perpetrator nevertheless still had or asserted those custody rights.

That custody, he doesn’t have a right to that anymore in a way. He’s thrown that away. [INV3]

Raising the children, you’ve shown that you’re not worthy of that. You see, if you can do such a thing, then you can’t teach your children anything. [INV18]

Perpetrators’ lack of genuine remorse, including a lack of willingness to answer questions about what happened and why, featured in many caregivers’ accounts, and was cited as an important reason for limiting contact, obtaining guardianship to protect the children, or for the children to limit or cease contact. For example, despite clear evidence, one father maintained the story that he thought that mother was a thief, which was detrimental for his relationship with the children. For other caregivers, it was the deliberate completion of the murder by father that informed their decisions regarding contact.

The aspect of remorse can be seen as part of a wider theme of taking responsibility. This also came up as relevant regarding family of the perpetrator: for example, one caregiver described that it was crucial that the family of the perpetrator clearly condemned the act. More broadly, several caregivers referred to examples of family members (often, but certainly not exclusively, from the perpetrator side) showing a lack of solidarity with the children. These examples ranged from a lack of interest to actively undermining placements, and included (a) children being ‘dropped’ by the family; (b) family members being unwilling to support children’s financial needs; (c) family of the perpetrator stimulating unhelpful and unregulated contact between perpetrator and the children; (d) family of the perpetrator telling a different story of the homicide – often blaming the victim – or laying an emotional claim on the children; and (e) family members not finding caregivers good enough for the children. Several of these elements came through in the following accounts:

On Thursdays, they sometimes stayed there for lunch, and they would come back almost in tears. ‘I'm not going to grandma and grandpa's anymore, they only talk about [Father] and how miserable he is.’ Yes, the word ‘mum’ or [mother’s name] was never mentioned by them again. [Father]'s brother said, ‘I’m not after having the children, I don't even want them.’ After the cremation, they didn't inquire about the children even once, literally not even once. And grandma and grandpa never asked them ‘How are you doing?’ They just crossed out my daughter's name from their birthday calendar with a pen, while a then ten-year-old said to grandma, ‘You could have just put a cross behind it.’ [INV18]

He would go to [the offender's family] every weekend. They would pick him up or I would drop him off. Until I found out […] that he was having conversations with his father over the phone. So they completely messed him up. He couldn't learn anything at school anymore, he couldn't do anything anymore … . [INV17]

Similar to situations with the perpetrator, caregivers recounted experiences of sometimes pushing back in these situations with children’s family members (e.g. proceeding with a funeral card made by the children, even though it contained typos which family members disproved of) or pushing through (e.g. a caregiver who was ostracised by their own family, from the perpetrator’s side, for looking after the children and condemning the murder, persevered).

Not every relationship was marred by conflict and some contact with family members was smoother than others. At times they suggested genuine solidarity and collaboration. An example was how four grandparents, from both sides of the family, helped each other to look after a baby in the acute aftermath of the killing. Another caregiver indicated that family from both sides were there for the child, and that she could rely on them. In other examples, the collaboration had not been there initially but developed rather as a result of working through conflict and ‘sticking with it.’

Caregivers appeared to build their stance also on personal norms about what should and should not be done. For example, some accounts implied that contact with at least some part of the biological family should be preserved, or that one shouldn’t withhold the children from the family of the perpetrator if it is clear that they condemn the killing. On the other hand, caregivers said that forcing children to have contact with a perpetrator is harmful, and that they should have a say in that matter, although a few conveyed that preserving some contact was logical.

These norms may put caregivers also in conflict with their own emotions. For example, this grandparent describes the difficulty that he would have with his grandchildren if they started showing affection for their father, who killed his daughter, although he does want to facilitate some contact to help questions being answered:

If those children eventually start showing affection towards their father, well then, I don't want it. Then those children don't need to live in my house anymore, so to speak. […] Then I think: Darn it. I've given them a sense of security and love. You see friends and acquaintances around you, they're traveling the world, but you just don't do that. You don't want to, because you consciously decided to look after the children. […] If the children eventually say: ‘Well, we want to see our father, and ask him: why did you do this? And why did you do this to us?’ we fully support that, that's not the issue. But I'm talking about the phase that will come after, when that father starts to have so much influence over those children again, when he's free, and he manages to sway them and they respond to his advances. That's what I struggle with. Because then I also feel guilty towards my daughter, that's the point. Because then I think: I can't subject my daughter to that. That thought, yeah, that's what I would struggle with. But my wife sees it differently, you know. She's a bit less troubled by that. [INV15]

### 3.3. Challenge 3: managing underprepared services

Although there was diversity in how caregivers experienced the support from services, many described their involvement and collaboration as a major challenge. They felt their own insights were ignored and the children not properly assessed, or that communication was lacking. In a few instances, a problematic initial engagement transformed into a helpful partnership over time, often because caregivers ‘stuck with it’ and committed to resisting certain directives until services changed their approach.

One of the main issues was that caregivers felt that their observations and assessment of the children and the family situation, including the behaviour of the perpetrator, weren’t taken seriously for a long time, sometimes until an external event proved them right. For example, one caregiver described that their concerns about contact with the perpetrator were only taken seriously after the perpetrator started cutting himself in court, with a razor blade he had smuggled in. In another family, the caregiver was also frustrated with the lack of consideration their concerns were given, and, similarly, felt that services ignored her views until the behaviours were directly visible to the service staff themselves:

She said, ‘You don't recognise him anymore, he's changed so much.’ I say, ‘He's still exactly the same, I see him at the trial, how he talks, how he behaves.’ So I wrote down: ‘I'm receiving a card now, the TBS clinic [compulsory forensic mental health clinic] has asked to stop sending cards to [Child]. My conclusion is that he is now going to send me a card, which will be so hurtful for me. But we'll wait and see,’ I wrote. I immediately sent that letter, because [Child’s] birthday was two days later. I received a card, and it said exactly what I had predicted. ‘I'll be released soon, my boy, and then I'm going to see you.’ But that fills me with fear. So I called immediately: ‘Did you receive my letter? Because I just received a card. And they were surprised. I said, ‘You know him so well, you've studied so much for that. I didn't need to study for that, you know. I see right through it.’ [INV21]

Caregivers described situations where staff did not properly assess how children were doing, for example just concluding that children were going well when they actually did not really engage in conversation during the assessment:

You put them all washed and ironed on the couch, and you say sweetly, ‘this is how we do it and we're very proper,’ and you know, I don't find it a thorough investigation. Even if those children were to do I don't know what here, you just get away with it. [INV12]

It came across as a box ticking exercise. Several caregivers felt that the workers were inexperienced, ill-equipped for the task and pressed for time, which, in combination with the frequent staff changes due to high turnover as well as some avoidance of responsibilities (one caregiver described services hiding behind other procedures not being finalised), led to decisions being based on extremely limited information:

Because if they had seen it, they would never have left [Child] with me. […] I could suddenly become enraged, and I wasn't stable at all. I was very nervous, constantly trembling on my legs and sweating. I couldn't handle [Child]'s crying at all. Well, in the report from youth services, or child protection, it says that I am so stable and reacted well. I thought: Girl, if only you knew. [INV21]

This caregiver continued to suggest that, instead, adequate assessment and subsequent support would have been more than welcome. In the last sentence of the quote above, the caregiver used the Dutch word ‘meisje,’ which translates to ‘girl’ but also something similar to ‘sweetie’ in the context of this sentence, conveying a young worker’s naivety. The caregiver in interview 12 mentioned above said something similar, although with a more negative tone, referring to some workers as a bunch of chickens (i.e. young and inexperienced):

I found the crisis foster care good, they were capable people. After that, we got all these chickens who had just started working and were all going to decide if we were good enough, I still don't think that's acceptable. [INV12]

This caregiver also recounted a stressful experience for the children related to the first prison visit, again conveying a disregard for caregivers’ many hours of observation of the children:

Also, the first visit of those children [to the perpetrator]. It was obviously a drama here, they were going to their father, everyone was upset, had stomach aches. [Brother1] ended up vomiting here in the parking lot, you wouldn't believe the stress there was. Then they had to go through all the security gates where [Child] became hysterical, well, it was a big drama. Well, there's a psychologist from the prison there … for father, of course, certainly not for the children. He thought it had gone fantastically well. Those children were beside themselves for two weeks, completely distraught. He came in crying like a wolf, sobbing, missing my sister so much, and he didn't know what had happened, completely playing into the victim role. So those children were extremely worried about daddy, and then I think that's not guidance at all, come on. [INV12]

As an additional aspect of the challenge, one caregiver outlined the time commitment their engagement with services took. Having to travel to various services, with some of them being over an hour away from home, took them away from the children for substantial periods of time, directly contrary to where they felt they should spend their time. Another said that engagement with services, various schools and the many legal procedures (court cases, notary interactions, etc.) was a daily job for them for 1.5 years after the homicide.

Caregivers also talked about the lack of communication from services, especially with regard to movements of the perpetrator (e.g. changes in parole conditions, imminent release, etc) that were relevant for the children or their family more broadly. They would occasionally seek out their own ‘informants’ in order to get updates, which they felt was important both from an emotional and safety perspective.

That man from the municipality, the one responsible for safety, knows the dates and he said, ‘Well, if I pass those dates on to the family, it will cause unrest.’ But it's the opposite. So I say, I always struggle when people start making decisions for us, instead of just stopping by and saying, ‘Hey, where can I provide you with information?’ [INV6]

At times, caregivers felt the (imprisoned) perpetrators’ needs were unjustly prioritised over the children’s, also in more trivial situations. For example, an overseas holiday for the children wasn’t approved by services:

They were very much oriented towards father; what does father want? Then I think, well, what does father want – does he still have a say then? [INV12]

Caregivers’ long-term friction with child protection or youth services eventually resolved in some instances. For example, one grandparent had been labelled as a criminal by youth services, which meant that, while the children stayed with him and his wife, they did not receive the financial support that they otherwise would have been entitled to. Eventually, this family was cleared, and they developed a good working relationship with the same services as a result. As a research team, we wondered whether this initial labelling also had a racist undertone, since people from Moroccan and Turkish descent, like this family, have traditionally been racially profiled in the Netherlands (see e.g. Andriessen et al., 2014) and we saw a contrast with the experience by white or autochthonous appearing families (see e.g. the quote from INV12 about the lack of proper assessment); this wasn’t mentioned as such by the caregiver however.

In case of the latter family, as well as others where conflict with services played a role, caregivers conveyed a sense of ‘sticking with it’ over time and persevering with their attempts to get services to see their side of the situation, with a few notable successes as a result. As one caregiver said:

But because that father is so incredibly difficult and has also engaged in several complaints procedures with youth services, youth services advised us: Essentially keep your distance, try to use youth services as a buffer. And that's what we're still doing, and thankfully it's going very well now. We had a very bad relationship with youth services because they went against our ideas. But thankfully that has completely changed, and they are fully on our side. [INV15]

This buffer function, or the lack of it when services were no longer involved, was mentioned by a few other caregivers as well. Services’ role as go-between could provide a helpful safeguarding mechanism, for example by intercepting inappropriate letters or advocating for the family or children.

Moreover, it should be noted that a few caregivers had very positive experiences with services from the start, for example, when workers quickly organised financial support, helped with navigating perpetrator contact requests, or were generally available for caregivers (‘If I need her then she’s there'). There was also mention of a worker who reinforced that a caregiver should look after herself, which is also relevant to the next challenge:

Because he also talked a lot with us, especially with me because he felt that I was ignoring my own needs, that my children came first. And he saw that things were actually going badly for me, and I had migraines. So he really hammered on it, saying, ‘Go see a doctor, go to the hospital, think about yourself for a change.’ So I really benefitted a lot from that. [INV19]

### 3.4. Challenge 4: enduring it, mentally and physically

The word ‘tough’ (‘zwaar’ in Dutch) came up multiple times when caregivers were describing their experiences. That it was tough to look after a child, even when you’re not the ‘grieving party’; a child that is so different from you and your own family. That it was tough to keep ‘six balls in the air’ especially when also having a child with special needs. That it was tough to look after the children and organise everything that had to be done for the funeral and otherwise related to the victim after the homicide. As came through in the previous three challenges, there was a sense of long-term sacrifice, mentally but also financially, physically, in terms of time and energy commitment and in terms of what daily life now looked like and would look like going forward. At the same time, caregivers conveyed motivation and a sense of purpose. Nobody said this exactly literally (although INV6 came very close), but caregivers’ accounts conveyed a paradox that could go something like this: ‘It’s too tough and I wouldn’t do it again. But I would do it again, for the kids.'

If we look back at what we've been through, I mean, you gain a lot of experience from it, you might think, we're never going to do that again, all that hassle around it, so to speak. But for [Child], we would do it all over again. [INV6]

Those who had been family of, or friends with, the victim described severe mental health consequences of the loss, and the taxing nature of looking after the children:

Well, on a Sunday, something happened, paint was spilled upstairs or something, and suddenly I just exploded. I started throwing things, I hit my husband, and it felt like that bomb had gone off inside me, that's how I felt. Yeah, my husband was so angry with me. But I was also just wetting my pants, you know? I was completely … I didn't understand myself. Well, my husband was angry, he didn't say anything to me all day, and the next morning he said, ‘Come on, let's go out for a bit.’ So I thought, ‘Oh, great, we’ll go out for a bit.’ Yeah, I just wanted to get outside. So hop in the car, he drove me to the doctor's, opened the door, and pushed me inside. And he went to talk to the doctor for a bit, and I thought to myself, ‘What's going on?’ Then the doctor said, ‘Now your husband can't handle it anymore, and you need to go to the mental health centre. I've already made an appointment, blah, blah, blah … ’ […] At first, I thought, ‘Ugh, all this nonsense, what am I doing here?’ On top of that, they put a box of tissues in front of me. I said, ‘Do you really think I'm going to cry here?’ But it really saved my life. [INV21]

One caregiver had nightmares of the children being killed by the perpetrator during a prison visit. Her partner expressed feeling guilty about not having picked up signals of coercive control before the homicide of their daughter. Several caregivers talked about their anxiety regarding future contact attempts by the perpetrator, as we saw in challenge 2. Multiple caregivers described experiencing strong grief for a long time, to the point of ‘going crazy’, which eventually improved due to mental health support (like the caregiver just above in interview 21 described). However, occasionally, caregivers also encountered situations where mental health support was not available or not accessed:

When this happens, people often don't realise that they need help. To be able to do that, you have to sit down, you have to talk about it, you have to seek help from a psychiatrist. But we didn't, we didn't seek anything. [INV17]

Several caregivers recounted heartbreaking conversations with the children. For example, this caregiver talked about the time just before the funeral, how she had to explain to the child that she would not be able to see her mother in the coffin:

I said, ‘Well, mommy isn't beautiful anymore.’ Yeah, I had to come up with something. ‘Yeah, mommy is still beautiful’ [she said]. Yeah, I found that quite difficult, how to explain all of that why she couldn't see her mother anymore, because, well, ‘Snow White also lay in a coffin, you could see her too,’ she said. [INV7]

Some caregivers also continued to be troubled by the question why the perpetrator had committed the murder, or why a previously good contact with the perpetrator had broken down.

Caregivers talked not only about emotional but also physical exhaustion, in particular the profound impact of caring for young children when you’re over 60 years old. This new role also brought about identity questions. For example, one caregiver described how she had never anticipated nor wished for having children herself. A grandparent explained it was challenging to make the transition from being a kind of sugar aunt who could not really make mistakes in raising the children, to being the primary caregiver, who definitely could. And another expressed concerns about possibly not living to see important milestones of the children (and being asked ‘Grandma, will you be there when i get married?’ by them), simultaneously having the role of a regular parent and not. She oscillated between worrying that they might be dead when the children would be 20 years old, and trying to live day by day since one cannot predict the future.

At the same time, caregivers conveyed a flipside to the burden and exhaustion: looking after the children provided them with purpose and energy. This caregiver expressed this combined, complex experience:

Yes, I think it's very important that the children experience safety and a loving environment, and you don't need to tell them that, they just feel it. Conversely, I think the children have given us an immense amount of positive energy. Of course, in the beginning, we were completely exhausted. I mean, by eight o'clock in the evening, we were already nodding off on the couch once they were finally in bed. Because it's not an easy ride when suddenly you have a seven-month-old baby and a two-and-a-half-year-old toddler, especially when you're already past the stage of caring for little children. To pick that all up again … So yes, it's quite a sacrifice you have to make. But it all went well, so those children simultaneously give you that positive energy. [INV15]

Other caregivers described the children as their motivational force, and that they drew strength from observing children’s positive development. Another pointed out that she might not have been alive anymore if it wasn’t for the grandchildren:

The guardian once said, it would have been better for you if the children were put in a foster home, then you could take care of yourself. I don't agree, I really don't agree. If I hadn't had those kids, I might not even be there. [..] You know, you still have something, how should one put it, something tangible from [Mother of the children]. I promised her that I will take care of her children and that kind of means … you go everywhere, you have to, you have to go shopping again, you have to go to the swimming pool, you have to go to the beach, yes, you name it, all you have to do in life again because those children are there. Otherwise you'd be downstairs [in the living room] and you wouldn't have to do anything anymore. I think that has been very good for us. [INV7]

A few caregivers also described how they drew meaning from being able to engage in the court process or supporting peers via a peer support association. Another aspect of meaning, which also transpired in the previous quote, came from maintaining a connection with the victim-parent. Sometimes this took the form of talking with the victim’s portrait, or it could be the promise to look after the children as mentioned above, or more implicitly a caregiver wondering ‘am I doing it right?’. This desire to do it right came through clearly in the following quote, which we include here as a final word:

And we only wanted one thing. To provide [Child] with … a … good home. A safe home. A loving home. That's what you do, right? [INV8]

## 4. Discussion

This study focused on caregivers’ accounts of raising children who had lost a parent due to fatal intimate partner violence in the Netherlands. Many, though not all, caregivers were relatives of the children. We conceptualised four related and ongoing challenges: bringing the children into the family fold; dealing with the perpetrator and relatives; managing underprepared services; and enduring it, mentally and physically. Sticking with their commitment to the children despite these challenges, caregivers also pointed to the potential for positive outcomes or turns of events, and recounted experiences of finding or making meaning.

The gravity and significance of the experience of caregivers in our study resonate with previous research. For example, Hardesty et al. (2008) described high levels of stress among caregivers, including due to family conflict and behaviour of the perpetrator, physical health concerns, and the diversity in children’s needs. Hardesty also wrote about present but quickly dwindling support in the aftermath of the homicide, and the importance of caregivers having a sense of agency in caring for the children, aligning with our findings that caregivers wanted to be more heard by services. The lack of long-term, skilled support also came through in Stanley and colleagues’ analysis of Domestic Homicide Reviews (2019) as well as in broader research on foster carers (e.g. Lotty et al., 2024) and grandparent-carers (e.g. Zuchowski et al., 2019, with the telling title ‘Convenient yet neglected’). However, our findings also suggested that, at times when families did feel supported or connected with others, this had an important positive effect, and recent literature points to initiatives that actively respond to families’ needs, such as the wrap-around service for bereaved children and their new caregivers in the State of Arizona (Websdale, 2022).

Our participants naturally wrestled with moral questions as to how to position themselves regarding what happened, other ‘stakeholders’, and what would be ‘best’ for the children. At times, there appeared to be a tension between their own norms or values regarding what should happen (e.g. that children should always have some contact with their family, or that a perpetrator loses all right to contact after a homicide that was not self-defence) and what children might want or need. It is likely that these two aspects also influence each other; for example, one’s values may colour whether and how one notices or interprets certain aspects of children’s behaviour, or children’s expression of their needs may shift one’s views on what is right. What is right or best is often a complex and nuanced question. While engagement and decision-making grounded in children’s rights and the evidence base on child and caregiver mental health and wellbeing after trauma is crucial (see e.g. Dooley et al., 2021; Landolt et al., 2017; Lundy, 2007; Reading et al., 2009), the specific characteristics of individual situations and frequent lack of resources necessitate a tailored approach. Being willing to adjust one’s personal moral stance or sacrifice elements of it because it allows a child to engage in a way that works for them, appears to point to a genuinely child-centred approach.

Exactly that, the centring of the children’s needs and interest, was what several caregivers were missing, in contact with the perpetrator, relatives, services, or others involved. In response, a throughline in our findings was the topic of commitment to a child and to ‘sticking with’ difficult family or service relationships for the sake of the children. Hardesty et al. (2008) talked about the potential value of approaches of ‘working together for the children’ that have been promoted for stepfamilies, including, for example, normalising the complexity of family relationships, the experience of conflicting emotions, and unresolved loss (see also e.g. Eikrem & Jevne, 2022). Our findings suggest that these approaches could indeed be valuable and that there may be potential for early mediation, also considering Harris-Hendriks et al.’s observation that there were few situations were family from both perpetrator and victim side were in contact without substantial conflict (Harris-Hendriks et al., 2000).

The caregivers in our study were navigating and performing multiple roles, as carer, advocate, negotiator, risk assessor, risk manager, and mediator, to name a few. Many of these roles were not officially acknowledged nor supported, leaving caregivers with the tasks of addressing knowledge gaps, learning new skills and, implicitly or explicitly, deciding how much effort and time to allocate to each of these roles. This speaks to the contrast between the largely informal nature of caregiving in the aftermath and the profoundly disturbing and disruptive nature of an intimate partner homicide.

In light of our findings, several practical implications for professionals working in child protection, youth services, placement services, mental health care, justice or other relevant agencies seem appropriate. It appears essential to shape conditions in which caregivers are taken very seriously with regard to their assessment of the children, their own mental health and wellbeing, their practical concerns, and their safety. In many cases, this needs substantial allocation of time and resources, including experienced workers (which would also decrease the chance of families having to deal with frequent turnover). Specifically, our recommendations are to:

- Ensure that caregivers have adequate professional and community support for their own mental health.
- Record caregivers’ own assessment of the children extensively and invest in in-depth complementary professional assessment of the children to obtain a thorough understanding of children’s wellbeing and needs.
- Involve caregivers in decisions regarding the children and keep them informed of developments (e.g. when a perpetrator is going to be on parole or released from prison), giving them and the children as much agency as possible.
- Engage in an early assessment of the existence of, and potential for, family conflict, and consider whether early mediation may mitigate such conflict, or whether caregivers and children would benefit from services playing a buffer role.
- Provide respite care (e.g. in weekends or evenings) if caregivers need this, especially considering caregivers who are grandparents of have other substantial caring duties or personal health considerations.
- Address financial and practical needs and concerns of caregivers pro-actively, rather than waiting for families to overcome all the formalities.
- Provide an open dossier where caregivers and children can easily take up a new episode of (mental health) care when needed.
- Ensure an inclusive, equitable, anti-racist and anti-classist stance in all interactions and decisions.

Over time, these elements may also feature as part of a formal needs assessment tool for caregivers who raise children bereaved due to parental intimate partner homicide. We come back to this point in the recommendations for future research below.

Several **study limitations** should be considered. The context of the larger mixed-methods study was such that participants engaged in clinical assessment of the children and themselves first, before the qualitative interview. Had we had multiple meetings with caregivers or no clinical assessment, the narratives would likely have had even more depth. Second, even though most caregivers looked back on a period of multiple years, there was substantial variation in this timeframe; in future studies it may be valuable to home in on a specific period in their caregiving journey. Third, we have touched on a few language aspects in the analysis to explain our interpretation of quotes. There were many more interesting language choices made by participants that we did not include for reasons of scope; we tended to the realist side of our critical realist stance here, simply because our results section was already sizeable, and we saw the four challenges as the core of our findings. Fourth, this study cannot and is not meant to be generalisable to all caregivers of children bereaved due to domestic homicide. Most likely, we spoke with a very specific subgroup; people who were willing to engage with researchers from a medical centre and keen to share their perspective. We hope that it provides starting points for further studies, which can over time evaluate commonalities and differences across settings, circumstances and researcher approaches. Finally, we collected our interview data 10 years ago, and some elements (e.g. ensuring children have a mobile phone with them) had a different connotation at the time than they have now. The emotional value of these narratives remains however, and the majority of the findings, including experiences with services, are just as relevant and timely (see e.g. Alisic et al., 2023 and Kurdi et al., 2024 for recent findings in Australia, the U.K. and Ireland).

Going forward, our findings and the field’s development inform a **potential research agenda**. Knowing that prevention efforts will be unlikely to fully reduce parental intimate partner homicides to zero, any research aiming to understand and improve children’ and families’ experiences in the aftermath is worthwhile. In recent years, substantial progress has been made with regard to the analysis of the content, procedures and impact of Domestic Homicide Reviews in England and Wales (see e.g. Rowlands, 2023; Rowlands & Cook, 2022; Stanley et al., 2019). This is leading to adjustments in policies and practices, and has motivated and allowed further research and monitoring, even though the researchers involved would probably be quick to point out flaws in both the system and the data (see e.g. Rowlands & Bracewell, 2022 and a note by Stanley et al that data collection was hampered by ‘inconsistencies in quality, nature and quantity of data’ that DHRs provide; p. 70).

For a research agenda, a diversity of methods and scale would be valuable; there is value in the deep understanding that can come from case studies as there is in large quantitative analyses comparing matched pairs (like Lysell et al., 2016, have done, for example). What then, can or should be the priorities? We propose four for discussion:

(A) *Evaluation of any current support approaches* (e.g. protocols, standard operating procedures, inclusion of children and their caregivers in DHRs in countries that have this system, etc.) will be very informative – not only for the relevant local context but also across localities and countries.
(B) *In-depth qualitative research* among all elements of children’s and caregivers’ ecosystem, including they themselves as well as professionals across a range of settings, family friends etc. Ideally, this would happen in a longitudinal set-up, with multiple engagements.
(C) *Quantitative research* where possible. There is very little opportunity for solid quantitative research in this domain (Lysell et al are an exception). It would be worthwhile to pool data available to analyse the impact of placement, contact, and other decisions that are fundamental for children’s lives. The combination of these evaluation, qualitative and quantitative studies (A-C) may also lead to the development of a tailored needs assessment tool for caregivers in this specific context.
(D) *Intervention studies*; it would be good to continue the work by colleagues like Soydas et al. (2023), who have analysed trauma-and grief-focused treatment (in an open trial format) for children bereaved due to parental intimate partner homicide, and Konijn et al (2020) who studied the potential of training caregivers in trauma-informed foster care. Existing evidence-based trauma and grief interventions will likely be helpful for caregivers and children alike, but, as pointed out above, may benefit from specific adjustments.

Placement and caregiving decisions in the aftermath of a parental intimate partner homicide reverberate in both children’s and caregivers’ lives throughout the years. The complexity of the challenges they face underscores the importance of concerted, continuing efforts to understand families’ needs and respond accordingly. The caregivers in our study showed remarkable perseverance and commitment; they and the children they look after deserve the best care and support possible.

## Data Availability

Due to the sensitive nature of the data, these are currently not available.
